# CAREGIVING, LIVING ALONE, AND OLDER WOMEN’S MENTAL HEALTH: A NUANCED VIEW AMID THE STRESS OF COVID-19

**DOI:** 10.1093/geroni/igae098.1160

**Published:** 2024-12-31

**Authors:** Yiwen Zhu, Justin Farmer

**Affiliations:** Harvard T. H. Chan School of Public Health, Boston, Massachusetts, United States; Harvard T.H. Chan School of Public Health, Somerville, Massachusetts, United States

## Abstract

Caregiving and living alone are potentially stressful psychosocial experiences for older adults, but prior findings are inconsistent: caregiving may be associated with prosocial benefits, and living alone is distinct from social isolation. In the context of heightened collective stress during COVID-19, we examined caregiving and living alone in 27,646 older female former nurses (mean age 67) from the Nurses’ Health Study II. We assessed whether caregiving and living alone were prospectively associated with multiple facets of mental health. In May 2020, participants self-reported personal caregiving roles (yes/no) and living arrangements (alone/with others). Caregivers reported their stress levels concerning common COVID-19-related caregiving tasks; all participants reported on social support. Seven mental health outcomes were assessed at 1-6 timepoints over one year, including depression, anxiety, perceived stress, loneliness, psychological well-being, social well-being, and gratitude. All analyses accounted for covariates including pre-pandemic mental health and multiple testing. Caregiving and living alone associated with higher distress and lower positive functioning. Nuanced perspectives emerged with respect to caregiving stress and social support: while stressed caregivers had worse mental health (e.g., OR depression =2.08[1.88-2.31]), caregivers not experiencing stress had better mental health (OR depression =0.74[0.67- 0.81]), in comparison to non-caregivers. Older women living alone with adequate social support showed comparable mental health to their counterparts who lived with others, while participants lacking support reported substantially elevated distress (e.g., OR depression =3.11[2.55-3.79]). We provided a more nuanced understanding of caregiving and living alone among older women; caregiving stress and social support may be promising intervention targets.

